# What Did Cantharis Cure?

**Published:** 1892-04

**Authors:** Frank Kraft

**Affiliations:** Cleveland, Ohio


### WHAT DID CANTHARIS CURE?

FRANK KRAFT, M. D., CLEVELAND, OHIO.

Some four or five months ago a lady came to my office suffering from great pain in the left side of the body, the attack, having set in some twenty-four hours before, was constantly increasing, and would culminate, as she said, in a violent paroxysm of pain so great that the doctors who have always been called in at such juncture would give hypodermics of Morphine until the pain was deadened. Pains of this kind had been known to her even as a school girl, though her married life was comparatively free until the last four years (now ætat. forty-one), after the birth of her first and only child. Since this event she has had these spells perhaps as frequently as once in three or four months. The lady from her own statement and a rather close oral examination is the picture of health; never an ache or a pain except when this paroxysm sets in. The doctors had variously diagnosed the trouble as gall-stone (in the left side) and as ovaritis. The history elicited was Bright's disease for the father and asthma for the mother, both now dead. She herself had had catarrh of the head in the worst form, and every year promptly from the 10th to the 12th of August hay fever claimed her as a victim. The catarrh had given way to local treatment of an eminent specialist last winter, but the hay fever she was not at all sure about.

The principal symptoms presented on this visit were a constant, cutting, jagging, "sprangly" pain in the left side, following very closely the ureter; gnawing pain in the "small" of the back that at times nearly "killed" her; and a constant teasing to pass water which would, when gratified, be but a few drops or a teaspoonful and be very painful.

Now what this lady wanted was something to keep off this attack of pain which she knew was coming, and she did not want either Morphine or Chloroform. Could that be done? I thought it could. I gave several powders of Cantharis$^{2m}$, with directions to take one whole powder at once, and dissolve another in the usual quantity of cold water, one teaspoonful of which solution to be taken immediately after the effort at urination. I asked to have brought to me on the morrow the urine voided until her return.

On the morrow the patient returned, reported no increase of pain, but still there was grumbling and growling there, and she wasn't quite content to trust the powders already given and taken. The urinary symptoms were gone. The Mason fruit-jar (one quart) was filled with a muddy urine, giving out the odor of turpentine, and depositing a sediment very nearly one inch deep, of a gritty, white substance. On chemical test, found it well loaded with albumen.

The sight of the urine and its deposit, and the explanation of the albumen rather shook the lady's faith in her perfect health, and she agreed when this paroxysm was done to put herself under a course of treatment. I gave another Cantharis powder, and a half-dozen blanks, and asked for another specimen of urine. The patient appeared duly, said she was perfectly well, no pain, no ache, and the urine when produced was clear as crystal.

So far the hypercritical reader has discovered that this patient suffered from renal colic, and that instead of Cantharis$^{2m}$ curing the case, it was simply a milder attack than formerly of renal colic, and nature itself threw off the morbific pain-producing products, and the lady recovered. And Cantharis$^{2m}$ had nothing to do with the case. Perhaps not. But let us go a little farther.

On the night of Aug. 19th, this lady called me to her house, and asked if I still believed it necessary for her to undergo examination and treatment, seeing that she had so promptly recovered, and had continued so well ever since. "Why, do you know, Doctor, that this is my hay-fever time, and it has never before failed to come within twenty-four hours of the 10th of August; but here it is the 19th, and I haven't had a touch of it, and don't feel the least bit like it." I was about to put in a modest claim for having kept off the annual visitant, when the whole cake was knocked into pi by—"but I took so much treatment last winter for my catarrh that I thought maybe that treatment also cured my hay fever, so to-day I called on the catarrh-doctor, and asked him if he had also intended to cure my hay fever when he was at work on my catarrh, and he said that that was just exactly what he had done!"

Between the hypercritical critic already referred to, and this specialist doctor, Cantharis$^{2m}$ in this case seems to be somewhere between the devil and the deep sea. "What did Cantharis cure?"

